# Site-specific methylation changes in the glucocorticoid receptor exon 1F promoter in relation to life adversity: systematic review of contributing factors

**DOI:** 10.3389/fnins.2014.00369

**Published:** 2014-11-21

**Authors:** Nikolaos P. Daskalakis, Rachel Yehuda

**Affiliations:** ^1^Traumatic Stress Studies Division, Department of Psychiatry, Icahn School of Medicine at Mount SinaiNew York, NY, USA; ^2^Mental Health Patient Care Center, James J. Peters Veterans Affairs Medical CenterBronx, New York, NY, USA; ^3^Laboratory of Molecular Neuropsychiatry, Department of Psychiatry, Icahn School of Medicine at Mount SinaiNew York, NY, USA; ^4^Department of Neuroscience, Icahn School of Medicine at Mount SinaiNew York, NY, USA

**Keywords:** NR3C1, glucocorticoid receptor, promoter methylation, exon 1F, adversity, psychiatry, psychopathology, development

## Abstract

There has been recent interest in epigenetics in psychiatry since it offers a means of understanding how stressful life experiences, in interaction with the genotype, result in epigenetic changes that result in altered gene expression, ultimately affecting the risk for mental disorders. Many studies focused on methylation of the glucocorticoid receptor exon 1F promoter following an initial observation that changes in this region could be modulated by the environment. This review examines all published studies that have attempted to measure methylation in this region using different techniques, several tissue types, populations at different behavioral state and stages of development. Methodological issues have been raised with the aim of attempting to understand methylation quantification and site of action. We propose that it is useful to examine whether methylation at specific sites within the promoter region may be particularly relevant to psychiatric vulnerability to stress-related outcomes.

Epigenetic plasticity is a mechanism through which environmental exposures influence genetic predispositions resulting in persistent alterations in gene expression and protein synthesis (Zhang and Meaney, 2010; Feil and Fraga, 2011). There has been recent interest in epigenetics in psychiatry since it offers a means of understanding how stressful life experiences, in interaction with the genotype, result in epigenetic changes that result in altered gene expression, ultimately affecting the risk for mental disorders (Tsankova et al., 2007; Yehuda and Bierer, 2009; Nestler, 2014).

Among multiple epigenetic modifications, DNA cytosine methylation has been most reliably studied in experimental and clinical settings (Olkhov-Mitsel and Bapat, 2012; Klengel et al., 2014). Studies attempting to understand stress-dependent developmental programming, have largely focused on promoter methylation of stress-regulatory genes, such as the glucocorticoid receptor (GR) gene, in association with vulnerability and resilience to psychiatric disorders (Daskalakis et al., 2013). The first of these studies examined the rat hippocampal GR exon 1_7_ promoter methylation showing an association with variation in maternal care the first week of life particularly at the nerve growth factor-inducible protein A (NGFI-A) binding sequence (Weaver et al., 2004). Soon, other methylation studies of the ortholog human GR promoter (GR exon 1_F_ promoter; GR-1_F_ promoter) emerged. This promoter also contains binding sequences for NGFI-A (two canonical and two non-canonical; Figure 1). In this paper, we present a systematic review of 16 studies that examined methylation in this region and reported methylation changes in the specific C—phosphate—G dinucleotides (i.e., CpG sites) in relation adverse experiences or adversity-related conditions (Oberlander et al., 2008; McGowan et al., 2009; Dammann et al., 2011; Perroud et al., 2011, 2014a,b; Tyrka et al., 2012; Conradt et al., 2013; Hompes et al., 2013; Melas et al., 2013; Martin-Blanco et al., 2014; Na et al., 2014; Romens et al., 2014; Van Der Knaap et al., 2014; Vukojevic et al., 2014; Yehuda et al., 2014b); Figure 2. More studies examined methylation in this region (Moser et al., 2007; Alt et al., 2010; Radtke et al., 2011; Mulligan et al., 2012; Steiger et al., 2013; Yehuda et al., 2013, 2014a; Rodney and Mulligan, 2014), but only the above 16 report methylation differences at a single CpG site resolution. It is already becoming clear that different studies use different methodologies, examine slightly different sub-regions, and accordingly, produce different findings with respect to directionality of the associations with stressful experience and stress-related illness (Figure 2). To date, most studies draw conclusions about whether the GR-1_F_ promoter is hyper- or hypo- methylated based on the average % methylation across several CpG sites. Upon careful review of the data we propose that it is equally useful to examine whether specific sites within the promoter region may be particularly relevant to psychiatric vulnerability to stress-related outcomes.

**Figure 1:**
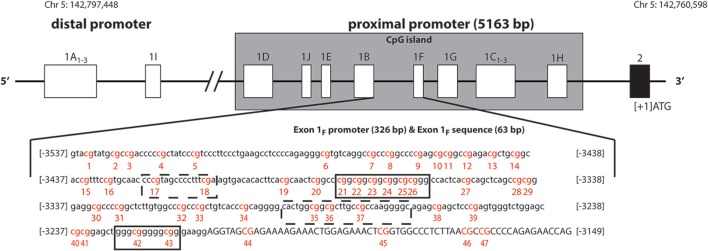
**Schematic representation of human glucocorticoid receptor gene (NR3C1) non-coding first exons according to Turner and Muller (2005), Sinclair et al. (2012), Steiger et al. (2013)**. The solid black line boxes with a number represent the different exons and the 5′–3′ orientation goes from left to right. The NR3C1 gene 5′ region is composed of multiple first exons: four in the distal promoter region (A_1–3_ and I) and ten (D, J, E, B, F, G, C_1–3_, and H) in the proximal promoter region located in a C—phosphate—G (CpG) island. The exon 1_F_ promoter (lower case) and exon 1_F_ sequence (uppercase) is illustrated. The numbering is relative to the start codon (ATG: +1), which is located 13 nucleotides downstream from the start of exon 2. The 47 CpG sites are in red and numbered. Boxes represent known or putative canonical (solid-lined box) and non-canonical (broken-lined box) NGFI-A–binding sites according to McGowan et al. (2009).

**Figure 2:**
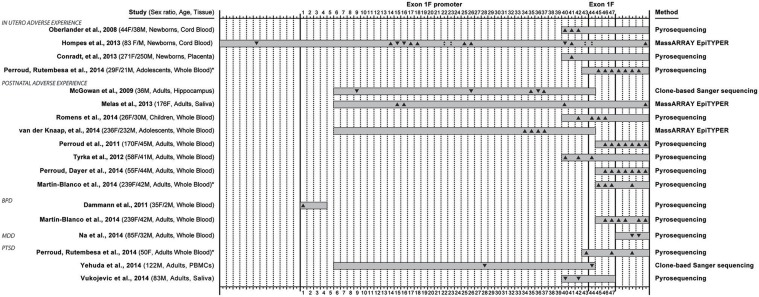
**Overview of 16 studies that examined methylation in the exon 1_F_ promoter and exon 1_F_ region of the glucocorticoid receptor gene (*NR3C1*), and reported methylation changes in specific C—phosphate—G dinucleotides (i.e., CpG sites) in relation to adverse life experiences (occurring *in utero* or early postnatal life) or conditions associated with adversity: borderline personality disorder (BPD), major depressive disorder (MDD) and post-traumatic stress disorder (PTSD)**. The 47 CpG sites in the exon 1_F_ promoter (sites 1–43) and exon 1_F_ (sites 44–47) regions are numbered according to Figure 1, while 12 sites upstream or 5 sites downstream of the region are not numbered. The coverage of every study is represented with a gray box and the direction of the site-specific findings is also depicted (▴ for hypermethylation, ▾ for hypomethylation, ▴▾ for both hyper- and hypo- methylation). The sex distribution, age group of participant at assessment and type of collected tissue are indicated in the left, while the method of quantitative methylation analysis used is indicated on the right. ^*^ Denotes the two studies (Perroud et al., 2014b and Martin-Blanco et al., 2014) that were included twice in this overview because they contained site-specific methylation data based on both adverse life experiences and adversity-related conditions.

## Promoter methylation

Heightened promoter methylation is typically associated with downregulation of gene expression, whereas intragenic methylation correlates with higher transcriptional activity (Jones, 2012; Moore et al., 2013; Yang et al., 2014). Methylation at promoter regions of highly expressed genes is often low contributing to the notion that promoter methylation is negatively associated with gene expression (Weber et al., 2007; Moore et al., 2013). Most of the studies in such hypomethylated genomic regions detect increases in methylation under pathologic conditions (e.g., Tan et al., 2013), but this could be also related to lack of methodological sensitivity to detect decreases in methylation. The transcriptional repression by methylation is thought to be mediated by blockade of transcription factor binding (Weber et al., 2007; Moore et al., 2013). The extent of promoter hypomethylation needed to enhance gene-expression, and conversely, the extent of hypermethylation required for reduction in gene expression is currently not known. In cancer research, and severe developmental disorders, large effects have been observed (Robertson and Wolffe, 2000; Bergman and Cedar, 2013). However, modest changes could have functional impact for example in psychiatric conditions if they are stable and produce meaningful changes in functional outcomes (Yehuda et al., 2013; Klengel et al., 2014). It is noteworthy, that even in cancer small percent changes in promoter methylation have been found to have great impact (Galetzka et al., 2012).

## GR-1_F_ promoter methylation

The human GR gene (*NR3C1)* is localized on chromosome 5q31-q32, contains nine exons (1–9), with the start codon located 13 nucleotides downstream from the start of exon 2. The 5′untranslated region (5′UTR) has 14 exon 1 splice variants (Figure 1, Turner and Muller, 2005; Steiger et al., 2013), all of which bear unique splice donor sites and share a common exon 2 splice acceptor site (Turner and Muller, 2005). Four of these alternative first exons (A_1−3_ and I) and their promoters are forming the distal promoter region of the gene (30 kb upstream of the start codon), while the other 10 first exons (D, J, E, B, F, G, C_1−3_, and H) and their promoters are forming the proximal promoter (5 kb upstream of the start codon, Figure 1), which comprises of a CpG island. The usage of the alternative exon 1 promoters, and the resulting participation of the alternative exon 1 splice variants in the mature GR mRNA, is tissue specific. The promoter usage is considered to eventually affect the expression of GR mature transcripts and protein isoforms, but the nature of this relationship is not known (Turner et al., 2010, 2014).

The GR-1_F_ is transcriptionally active in hippocampus, B lymphocytes and innate immune cells (but not in T lymphocytes or monocytes) (Turner and Muller, 2005). GR-1_F_ promoter (326 bp) and exon 1_F_ (63 bp) contain 47 CpG sites (Figure 1). Methylation of the GR-1_F_ promoter region associated, in neonatal cord blood, with maternal depression during gestation (Oberlander et al., 2008) and, in adult offspring hippocampus, with childhood abuse (McGowan et al., 2009). We have recently observed lower peripheral blood mononuclear cells' (PBMCs) methylation of the same promoter region in adult combat exposed veterans with PTSD compared with combat exposed controls (Yehuda et al., 2014b). The small % changes in GR-1_F_ promoter methylation had a functional impact since it was associated with endocrine functional outcomes (Yehuda et al., 2014b).

## Technical considerations on methylation analysis of GR-1_F_ promoter region

### Method

DNA methylation analysis involves bisulfate treatment of the DNA as an initial step. Bisulfite treatment describes the conversion of all unmethylated cytosine residues to uracils (deamination) in the presence of NaOH and sodium bisulfite, but leaves the methylated cytosine residues intact. Thus, sequencing of the treated DNA after amplification of the target region allows the analysis of methylation, by determining the ratio of cytosine to thymine, at a single CpG site level (Tost and Gut, 2007; Zhang et al., 2009).

Quantitative measures of DNA methylation patterns are essential in the context of disease. Given the heterogeneity in methylation between cells, Sanger sequencing of bisulfite-treated samples alone is not sufficient for quantitative analysis of methylation status of the target region. To overcome this, cloning of the region of interest (usually 300–500 bp) followed by sequencing of individual clones is needed (Olkhov-Mitsel and Bapat, 2012). Alternatively, the use of other sequencing methods with improved quantitative resolution does not require the expensive, time-consuming and laborious clone-based methylation analysis. Pyrosequencing gained popularity the recent years as an accurate and reliable approach for methylation analysis of short DNA stretches (usually <150 bp). Bisulfite treated DNA is first amplified and then, since one of the amplification primers used is biotinylated, a single strand is isolated. Finally, with the use of a pyrosequencing primer, the purified single strand is subjected to a pyrosequencing reaction where single nucleotides are incorporated sequentially and generate light that can be detected and quantified (Tost and Gut, 2007).

No direct comparisons have been made with respect to Sanger sequencing and pyrosequencing with respect to the GR-1_F_ promoter methylation analysis. Methylation in this region has been analyzed by some using clone-based Sanger sequencing (McGowan et al., 2009; Yehuda et al., 2014b) and by others using pyrosequencing (Oberlander et al., 2008; Dammann et al., 2011; Perroud et al., 2011, 2014a,b; Tyrka et al., 2012; Conradt et al., 2013; Martin-Blanco et al., 2014; Na et al., 2014; Romens et al., 2014; Vukojevic et al., 2014). More recently, the Matrix-assisted laser desorption ionization time of flight mass spectrometry (MassARRAY) provided another method for quantitative methylation analysis of long DNA sequences (usually >600 bp), based on fragmentation by base-specific cleavage and subsequent analysis of the cleavage products (Claus et al., 2012) and three studies in this review have utilized it (Hompes et al., 2013; Melas et al., 2013; Van Der Knaap et al., 2014).

### Tissue type

Methylation patterns at transcriptional regulatory elements, which contain transcription factor binding sites, are tissue-specific and cell-type specific, apart from development-, individual-, or disease- specific (Zhang et al., 2013; Ziller et al., 2013). Tissue-type and cell-type specific methylation changes occur at evolutionary conserved sequences (Zhang et al., 2013). The tissue-specific methylation differences, in an order of magnitude, are larger than the cell-type specific differences (Ziller et al., 2013). Promoter methylation seems to participate more in tissue differentiation, whereas enhancer methylation in cell-type differentiation (Zhang et al., 2013). DNA in the under review studies was extracted from a wide range of tissue-types (blood, brain, placenta, saliva) involving many cell-types, suggesting that a big part of variance between studies could be related to this choice.

In psychiatry, there is special interest on peripheral tissues, more often blood, in methylation studies because peripheral tissue is more readily accessible. Using peripheral methylation as a surrogate to brain methylation has been supported by two types of data: first, causal biological mechanisms of the condition studied can affect peripheral methylation, in an independent way to brain methylation, providing useful peripheral biomarkers; second, condition-related methylation patterns in some loci can be the same in the periphery and brain revealing common condition-related epigenetic reprogramming giving clues on developmental origins of the condition (Aberg et al., 2013).

### Sub-region of interest within the GR-1_F_ promoter

When observing the studies under review in chronological order, several trends in the field can be observed (Figure 2). Initial publications have an extremely high influence on the choices of regions of interest in subsequent studies. Oberlander et al. (2008) choice focused on methylation at one canonical NGFI-A binding site sequence was motivated by previous work (reviewed above) in rats by Weaver et al. (2004) on the rat ortholog sequence. The technique that Oberlander et al. chose (i.e., pyrosequencing) limited the sequence length (105 bp) which trucated the number of CpGs under investigation to eight, four in exon 1_F_ promoter and four in exon 1_F_ (another five sites were measured from the downstream region) from 47 potential sites. This first original human publication investigating the effects of prenatal maternal mood on newborn cord blood on methylation, affected the choice of technique and region for two studies examining similar prenatal factors (Conradt et al., 2013; Perroud et al., 2014b), but also seven studies that examined other questions entirely (Perroud et al., 2011, 2014a,b; Tyrka et al., 2012; Martin-Blanco et al., 2014; Romens et al., 2014; Vukojevic et al., 2014).

Similarly, the choice of McGowan et al. (2009) to study 39 CpG sites (38 in exon 1_F_ promoter, 1 in exon 1_F_) containing 4 NGFI-A binding sites (2 canonical and 2 non-canonical, Figure 2) in association with childhood abuse on adult hippocampal methylation has influenced the choice of another study of early adversity (Van Der Knaap et al., 2014) and a study of combat-related PTSD (Yehuda et al., 2014b). Two recent studies using MassARRAYs reported methylation for even larger regions (Hompes et al., 2013; Melas et al., 2013).

### Stability

There is an emerging dialectic between the idea that epigenetic changes are enduring enough to persist through gamete mitosis and meiosis and the idea that epigenetic marks may undergo observable changes in response to the environment throughout life (Bergman and Cedar, 2013). The apparent discrepancy between these two ideas is partly resolved by the idea that the nature of epigenetic changes may in part depend on regional specificity (gene promoter vs. gene body) and sub-regional specificity (site-specific effects).

## GR-1_F_ promoter site-specific methylation

From the 16 studies reviewed (Oberlander et al., 2008; McGowan et al., 2009; Dammann et al., 2011; Perroud et al., 2011, 2014a,b; Tyrka et al., 2012; Conradt et al., 2013; Hompes et al., 2013; Melas et al., 2013; Martin-Blanco et al., 2014; Na et al., 2014; Romens et al., 2014; Van Der Knaap et al., 2014; Vukojevic et al., 2014; Yehuda et al., 2014b), four studies showed site-specific effects in offspring based on maternal mood during gestation [anxiety (Hompes et al., 2013), depression (Oberlander et al., 2008; Conradt et al., 2013; Hompes et al., 2013), PTSD (Perroud et al., 2014b)], six studies investigated the site-specific effects of adversity occurring in the period from birth to adolescence [abuse (McGowan et al., 2009; Perroud et al., 2011, 2014a; Tyrka et al., 2012; Martin-Blanco et al., 2014; Romens et al., 2014; Van Der Knaap et al., 2014), low parental care (Tyrka et al., 2012), parental death (Melas et al., 2013), parental loss (Tyrka et al., 2012) and other early stressful life events (Van Der Knaap et al., 2014)], and four studies detected differences in subjects meeting criteria for disorders associated with life adversity [BPD (Dammann et al., 2011; Martin-Blanco et al., 2014), MDD (Na et al., 2014), PTSD (Perroud et al., 2014b; Vukojevic et al., 2014; Yehuda et al., 2014b)]. There was further variation across studies based on the age (neonate, adolescent, adult) and sex distribution (male only, female only, mixed) of the sample used.

While the majority of studies detected hypermethylation in the specific CpG sites, there were sites where hypomethylation was detected demonstrating a general positive association of adversity with methylation of this region. However, the lower rate of detected hypomethylation in association with adversity might be also related to a “floor effect” due to low overall methylation levels in this region, which make difficult to statistically prove lowering of methylation. Potential false negative detection of hypomethylation needs to be investigated further in large studies especially since it can also have effects in downstream expression and functional endocrinology (Yehuda et al., 2014b) so as hypermethylation (McGowan et al., 2009).

If different adversity factors and associated conditions can affect the methylation of different CpG sites in opposing directions, it remains to be explored what is the outcome of the opposing methylation changes for gene expression. Only two of the 16 studies provided data on gene expression in relation to site-specific methylation in GR-1_F_ promoter. We have reported a negative correlation of GR-1_F_ expression with methylation at site 28 (Yehuda et al., 2014b), which was one of the two sites associated with PTSD. Vukojevic et al. observed an inverse correlation of GR expression with methylation at site 42 in healthy young adults, but they did not report expression data in the sample, where they observed the association with PTSD (Vukojevic et al., 2014).

Furthermore, while there were CpG sites for which multiple studies were in agreement (CpG 35, 37, 40, 41, 42, 43, 44, 45, 46, and 47), but there were also sites with disagreement (CpG 15, 16, 22, 23, 26, 36, 40, 42, 43, and 44). Factors that could potentially account for the disagreements are different time window in which adversity acted (1st -2nd - 3rd trimester of pregnancy, postnatal period and childhood, adulthood), differences in tissue type, subject's sex and age (at assessment).

## Possible trangenerational effects of parental trauma exposure and symptoms on GR-1_F_ promoter site-specific methylation

**Figure 3:**
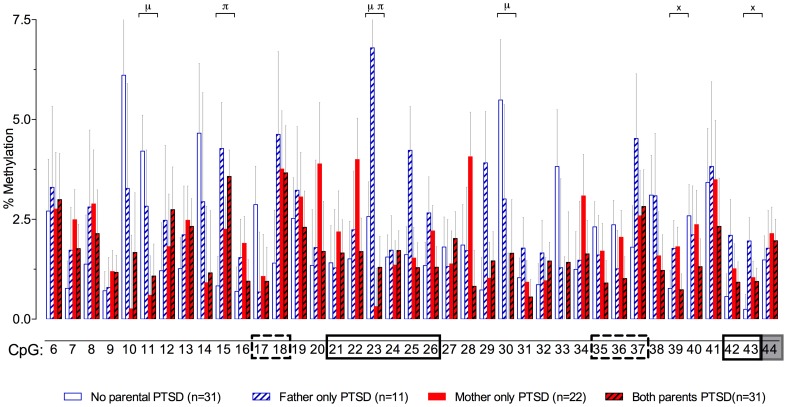
**Percent methylation at each of 39 C-phosphate-G dinucleotides (i.e., CpG sites) of the glucocorticoid receptor gene (*NR3C1)* exon 1_F_ promoter (sites 6–43) and exon 1_F_ region (site 44) analyzed by cytosine methylation bisulfite mapping (clone-based Sanger sequencing) using peripheral blood mononuclear cells (PBMCs) DNA, in a population of offspring described in our previous publications (Lehrner et al., 2014; Yehuda et al., 2014a)**. The 39 CpG sites accessed in the study are numbered as in Figure 1. The represented data (mean ± s.e.m.) are based on a multivariate Two-Way analysis of covariance with maternal and paternal post-traumatic stress disorder (PTSD) as fixed factors and parental Holocaust exposure, age, smoking history, and PBMC-type as covariates. μ, significant maternal PTSD effect. π, significant paternal PTSD effect. x, significant maternal by paternal PTSD interaction effect. Significance was set at *p* < 0.05. Boxes represent the location of known or putative canonical (solid-lined box) and non-canonical (broken-lined box) NGFI-A binding sites, according to McGowan et al. (2009), and a gray box represents the location of the beginning of exon 1_F_.

In a recent study in Holocaust survivor offspring and demographic controls (Yehuda et al., 2014a), we wanted to identify effects of parental Holocaust exposure or exposure-related symptoms (i.e., PTSD) on GR-1_F_ promoter methylation in order to find epigenetic marks associated with glucocorticoid dysregulation (Bader et al., 2014; Bierer et al., 2014; Lehrner et al., 2014) in this population at risk for PTSD. Paternal PTSD, only in the absence of maternal PTSD, was associated with higher levels of GR-1_F_ promoter methylation, while offspring with both maternal and paternal PTSD displayed the lowest level of methylation. The relatively small differences in methylation were associated with differences in GR-1_F_ expression and functional endocrinology (Yehuda et al., 2014a). A site-specific analysis presented here (Figure 3), reveals that there are sites with significant effects of maternal PTSD (CpG 11, 23 and 30), paternal PTSD (CpG 15 and 23) or their interaction (CpG 39 and 43).

Exposure to trauma in parents has been also linked to an increased risk for child abuse and maltreatment in offspring especially in the presence of maternal or paternal PTSD (Yehuda et al., 2001; Yehuda and Bierer, 2008; Palosaari et al., 2013). It is difficult to disentangle effects that reflect trauma-related transgenerational inheritance from early rearing influences, including childhood traumatic events, experienced as a consequence of having trauma-exposed or symptomatic parents. We have previously suggested that part of the phenotype in Holocaust offspring is the recollection of perceived emotional abuse or neglect, whereas recollections of sexual abuse and physical abuse or neglect may be relatively independent risk factors for PTSD in Holocaust offspring, as in persons who develop PTSD without having traumatized parents (Yehuda et al., 2001). Phenotypic clustering of Holocaust offspring demonstrated an association of paternal, but not maternal, PTSD with childhood trauma and abuse and increased GR-1_F_ promoter methylation (Yehuda et al., 2014a).

## Conclusions

Because the study of epigenetics in neuropsychiatry is relatively new, many fundamental questions are just beginning to be answered. It is not clear whether epigenetic marks are equally stable across all genes and all gene regions since some epigenetic marks have been shown to persist across generations (Gapp et al., 2014), while others have demonstrated change in response to psychotherapeutic interventions (Perroud et al., 2013; Yehuda et al., 2013). At the current time, complicating the discussion about the stability of epigenetic marks is that the reliability of the assessment of epigenetic marks such as methylation is not fully explored making difficult to identify the rate of potential stochastic epigenetic phenomena (Nestler, 2014). Thus, even in studies that assume epigenetic marks to be stable, there is value in performing multiple assessments of the same sample, or different samples from individuals within short periods of time. Such studies have been lacking, but will be very informative if performed in the near future.

### Conflict of interest statement

The authors declare that the research was conducted in the absence of any commercial or financial relationships that could be construed as a potential conflict of interest.
